# Characterization of Visual Scanning Patterns in Air Traffic Control

**DOI:** 10.1155/2016/8343842

**Published:** 2016-04-07

**Authors:** Sarah N. McClung, Ziho Kang

**Affiliations:** ^1^School of Electrical and Computer Engineering, University of Oklahoma, 110 W. Boyd Street, Devon Energy Hall 150, Norman, OK 73019-1102, USA; ^2^School of Industrial and Systems Engineering, University of Oklahoma, 202 West Boyd Street, No. 116, Norman, OK 73019, USA

## Abstract

Characterization of air traffic controllers' (ATCs') visual scanning strategies is a challenging issue due to the dynamic movement of multiple aircraft and increasing complexity of scanpaths (order of eye fixations and saccades) over time. Additionally, terminologies and methods are lacking to accurately characterize the eye tracking data into simplified visual scanning strategies linguistically expressed by ATCs. As an intermediate step to automate the characterization classification process, we (1) defined and developed new concepts to systematically filter complex visual scanpaths into simpler and more manageable forms and (2) developed procedures to map visual scanpaths with linguistic inputs to reduce the human judgement bias during interrater agreement. The developed concepts and procedures were applied to investigating the visual scanpaths of expert ATCs using scenarios with different aircraft congestion levels. Furthermore, oculomotor trends were analyzed to identify the influence of aircraft congestion on scan time and number of comparisons among aircraft. The findings show that (1) the scanpaths filtered at the highest intensity led to more consistent mapping with the ATCs' linguistic inputs, (2) the pattern classification occurrences differed between scenarios, and (3) increasing aircraft congestion caused increased scan times and aircraft pairwise comparisons. The results provide a foundation for better characterizing complex scanpaths in a dynamic task and automating the analysis process.

## 1. Introduction

Air traffic controllers are considered to have a highly stressful occupation due to the weight of their responsibilities and the constant expectation of their faultless performance [1, 2]. They monitor aircraft, communicate with pilots, and solve conflicts that threaten either loss of separation (LOS) of a minimum allowed distance between aircraft or wake turbulence [3]. Since 1980, industrial air traffic has averaged over 5% growth each year [4] and continues to steadily rise [5], causing ATCs to experience more difficulty with their tasks [6, 7]. Each ATC is assigned a sector in space and as the aircraft traffic increases, the sectors become more crowded [6]. Overload and scenario difficulty has been shown to decrease ATC performance [8], and since more aircraft cause higher probability of errors and ATCs are required to make no mistakes, it is highly important to aid ATCs by providing insight to efficient training methods and utilizing as much automation as possible.

**Figure 1:**
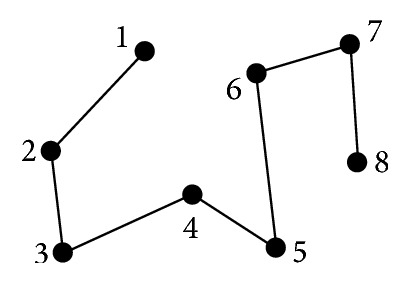
Scanpath example.

Previous research verified that one way to develop efficient training programs is by allowing novices to view expert ATC visual scanpaths to teach the novices the highest performing scanning strategies at the quickest rate [9, 10]. This method was appropriate because monitoring eye movements can aid in understanding user intent [11]. A scanpath is a sequential eye movement across a display [12–14], as depicted in Figure 1. The points represent eye fixations and the lines represent saccades, which are movements between fixation points [15]; the scanpath moves sequentially from point 1 to point 8. Eye tracking devices, such as those under monitors, have been used to successfully record, observe, and analyze visual scanpaths to improve computer interfaces [16, 17]. Because research has shown that understanding ATC cognitive processes is useful [18, 19] and observing scanpaths is a suitable method [20], automating detailed characterization of scanpaths would also be valuable to provide effective training techniques. If scanpath characterization can be accurately automated, expert ATC scanpaths can be collected, recorded, and characterized for novices to watch for deeper understanding. Additionally, novices can receive more feedback on their own scanpaths while running simulations to test the efficiency of their strategies as well as their performance.

However, there is a lot of uncertainty when attempting to characterize scanpaths. According to ATC linguistic inputs, scanning strategies can be conceptually described as being circular, linear, trajectory, regional, augmented, proximity-based, or density-based [9, 21], but realistically identifying a scanpath into only one of the listed categories is unlikely due to overlap caused by their elementary definitions. Therefore, the challenge exists in attempting to correctly map the ATC self-reported strategies, or patterns, to each of their actual scanpaths. This is difficult because scanpaths grow in complexity with time and become highly difficult to classify.

**Figure 2:**
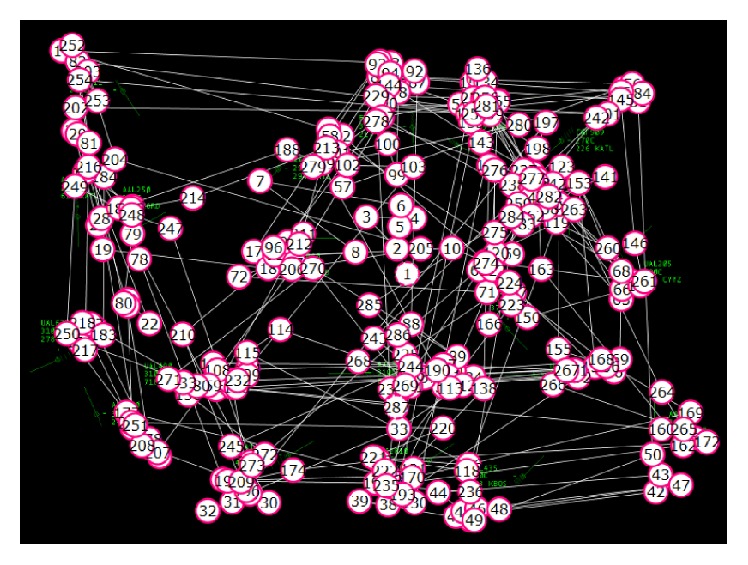
Example of a real scanpath overlaid on a static display of 1.5-minute duration.

Figure 2 shows a scanpath of 1.5-minute duration in which correct classification into the strategies provided by the ATCs would be unlikely. In addition to finding multiple overlapping patterns, the patterns can be incomplete, chaotic, or ambiguous.

**Figure 3:**
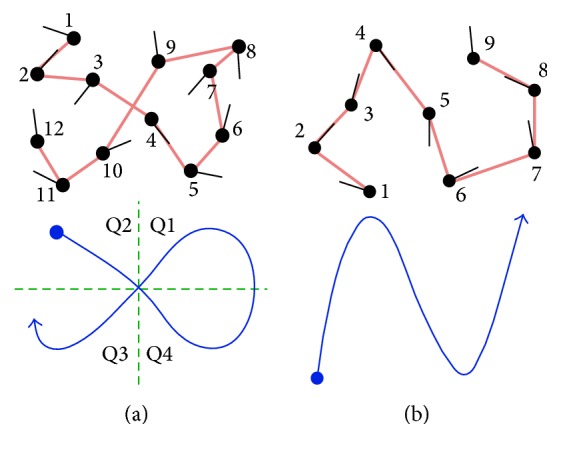
Examples of scanpaths with corresponding simplified patterns. The scanpaths overlaid on aircraft are shown above with saccades in red and the interpreted patterns are shown below in blue. (a) Circular, linear, and regional patterns can be identified. (b) Linear, trajectory, and density-based patterns can be identified.

Another major concern that causes classification uncertainty is that some scanpaths appear to cause a pattern that was not intended. Most expert ATCs intend to use a particular strategy, but extracting exactly what they intended from the data can be difficult. For example, an ATC can intend to use a circular scanpath, but it cannot be identified by the eye tracking data alone because of constant back-and-forth comparisons between aircraft and lack of a complete circle. The circle can switch directions several times from clockwise to counterclockwise in semicircles and can be interrupted by comparisons that cause the ATC to look across the screen in linear motions. When observing the plotted data, it is possible that the intended circular scanpath can be classified instead as mixed between linear and augmented. To visualize this issue, Figure 3 demonstrates additional fictional examples of basic scanpaths with their corresponding shapes. The numbers show the order the aircraft were viewed in, the thick red lines show the saccades, and simplified representations are below in blue. Choosing one pattern to label (a) and (b) from the many options available unfortunately relies on a judgement call, especially since the patterns are not exclusive. Scanpath (a) can be argued to be circular from fixations 3 to 10, linear from 1 to 8 or 5 to 12, or augmented from 1 to 12 moving from quadrants Q2, Q4, Q1, and then Q3. Scanpath (b) can be linear, trajectory, or density-based from fixations 1 to 9. Similar issues frequently occur when viewing ATC scanpaths. The patterns require thresholds and possibly hierarchical order to determine which strategy was dominant.

Other issues to consider are the level of influence on a scan from the number of aircraft, the difficulty of the scenario, and the spatial layout of the aircraft. Overall, the type of scan depends on the ATC's strategy and level of influence from the mentioned variables which often leads to multiple incomplete, local, or unclear patterns that prove highly challenging to characterize. For example, in [9], circular is defined as observing circular motions in the scanpath; however the details describing circular motions were not defined. Therefore, it is possible to have human judgement bias during interrater agreement if specific procedures to characterize and classify the scanpaths are not developed.

In order to begin addressing these problems in a simplified manner, consider limiting the scanpath patterns to only being circular, linear, and mixed as provided in Table 1. In this research, only circular and linear patterns were searched for because they are the simplest strategies to witness and most frequently used by expert ATCs [21] and depend only on scanpath shape. Ideal examples include counterclockwise, clockwise, and spiral for circular patterns and horizontal, vertical, and diagonal for linear patterns. The realistic examples are representations of the ideal examples; the mixed pattern uses both circular and linear movements to complete the scan, so there is no ideal form. The realistic examples are fictional, overlaid on a low congestion scenario of 12 aircraft to conceptually demonstrate the categories. Note that patterns can have a combination of their characteristics such as the second circular and linear examples that change direction before completion.

Due to the different possible interpretations and the high magnitude of complexity, scanpaths are difficult to classify into the patterns provided by the expert ATCs. Although some ideal and realistic examples are provided, there is still a challenge in mapping each scanpath to those selected patterns. Patterns are described conceptually, but there are no given thresholds or mathematical representations to confidently identify them. For example, how is a linear movement mathematically defined from an ATC verbal description (such as moving left to right while zigzagging [21])? Moreover, if linear movements are successfully represented in mathematical form, how are realistic scanpaths analyzed given that they do not move in perfect or predictable ways? Many conditions need to be considered, such as the random fluctuations that occur in scans including back-and-forth movements to previous aircraft. Although many algorithms were developed to compare and analyze visual scanpaths [12, 22–32] and their capabilities and limitations are provided in detail in [12], the methods were limited to comparing scanpaths but not mapping the visual scanpaths to strategies verbalized by the experts.

Naturally, it is difficult to develop mathematical models or algorithms based on verbal descriptions provided by the ATCs. The descriptions require more depth; after refining them, it may be possible to divide the scanpaths into patterns that can be confidently classified based on certain criteria. Pattern identification requires thresholds that address points of deviation, the percentage of aircraft viewed following a given pattern, and methods to correctly identify the pattern. Otherwise, many patterns can be claimed to be used in a scan although only one is dominant and intended by the ATC. If terminologies and procedures were predetermined and applied in a systematic manner, then more consistent and in-depth discoveries could be reached. Therefore, the purpose of this paper is to (1) introduce new terminology, (2) apply filtration methods to scanpaths to simplify their representation before judgement, (3) provide procedures that behave as a conceptual framework for raters during pattern classification, and (4) apply the proposed procedures to characterize scanpaths and compare their results across scenarios with different number of aircraft. This work is meant to ease characterization of scanpaths, increase characterization accuracy, and contribute to future automation of scanpath characterization.

## 2. Proposed Methodology

### 2.1. Terminology for Identifying Visual Scanning Strategies

New terminology is introduced in order to filter out the complex scanpaths into manageable forms that can lead to representations of the strategies provided by ATCs. Assume there are *N* number of aircraft present on the radar display for the definitions provided below:

(i) *Raw scanpath*: the raw scanpath is the entire scanpath including all fixations and saccades from the beginning to the end of the task being carried out by the ATC to solve all conflicts in the scenario.
(ii) *Global scan*: a global scan is a complete observation of all *N* aircraft.
(iii) *Local scan*: a local scan is an observation of a group of aircraft with possible comparisons, where the group contains 2 to (*N* − 1) aircraft.
(iv) *Comparison*: a comparison occurs when aircraft are consecutively scanned at least twice after already being viewed once before for a total of three or more observations until moving on to different aircraft.
(v) *Initial global scanpath (IGS)*: the IGS is the first complete observation of all *N* aircraft on the display. It includes all fixations and saccades that occurred until all aircraft were visited.
(vi) *Extracted IGS*: the extracted IGS is the IGS with comparisons removed, or filtered out; it applies the initial filter to the IGS.
(vii) *Fundamental IGS*: the fundamental IGS is the IGS without local scans; it applies the most intense filter and extracts the simplest form of the IGS. It displays the order each aircraft was viewed during the IGS.

During a global scan, if all *N* aircraft are visited, then the following eye fixation starts the next possible global scan. In a raw scanpath, there can be multiple global scans or none if all the aircraft were never viewed. Similarly, a global scan can include multiple local scans or none, and a local scan can include multiple comparisons or none. Particularly, they exist as subsets of each other as shown below:

 (Raw Scanpath) *⊆* (Global Scans) *⊆* (Local Scans) *⊆* (Comparisons).

**Figure 4:**
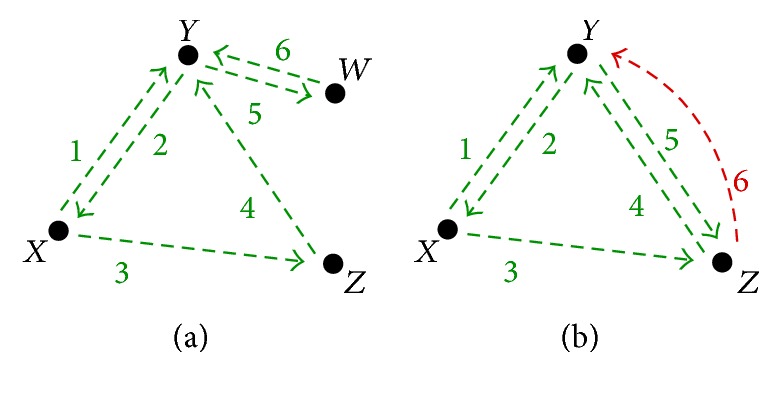
Local scan without and with a comparison. The black dots represent aircraft named *W*, *X*, *Y*, and *Z*. The numbered arrows indicate the order in which the aircraft were observed. Green indicates a local scan and red indicates a comparison. (a) Local scan between *W*, *X*, *Y*, and *Z* with no comparisons. (b) Local scan between *X*, *Y*, and *Z* and comparison between *Y* and *Z*.

The concept of local scans and comparisons is derived from the definition of visual groupings [33], or a significant amount of transitions between aircraft. Comparisons are made during conflict resolution between aircraft. When a comparison is finalized by an ATC moving on to different aircraft, the ATC can either return again to the same aircraft to perform another comparison, compare a different group of aircraft, or continue scanning. A comparison is always a local scan, although local scans are not always comparisons if the aircraft are merely scanned without repetition.  Figure 4 illustrates the difference between a local scan and comparison; (a) shows a local scan between *W*, *X*, *Y*, and *Z* with no comparisons while (b) shows a local scan between *X*, *Y*, and *Z* with a comparison beginning during the sixth movement between *Y* and *Z* shown in red. Note that the aircraft group shown (*W*, *X*, *Y*, *Z*) are only 4 out of many more aircraft on a radar display; thus viewing them does not complete a global scan.

Only the IGS was analyzed for number of comparisons and scanpath pattern because there are less chances of local scans and comparisons during that time. After the IGS is completed, all the aircraft have been viewed and the remaining time is most likely spent on conflict resolution which does not require additional global scans. Individual ATC scanning strategies are most likely to be witnessed during the IGS with more ease and clarity compared to the rest of the scan.

However, the problem remains that the IGS usually consists of multiple local scans with comparisons causing it to still be difficult to classify. Applying filters to the repetitive movements eases the classification process; therefore the extracted and fundamental IGSs were used. The extracted IGS represents how raters should naturally observe scanpaths while watching and judging scanning strategies; it disregards comparisons between aircraft but has to consider local scans. The fundamental IGS is the most simplified representation of a scanpath; as previously mentioned, it disregards local scans which implies excluding comparisons as well. It has *N* number of fixations and (*N* − 1) number of connections between fixations. The connections between the fixations are not necessarily saccades since they merely show the path to the next viewed aircraft by the ATC without considering any repeat observations to previous aircraft. Particularly, the aircraft are numbered from 1 to *N* in the order they were fixated on; then connections are drawn sequentially between the fixations.

Once the extracted and fundamental IGSs are obtained, their patterns can be classified. The scanpath strategies consist of seven known pattern categories: circular, linear, trajectory, regional, augmented, density-based, and proximity-based categories [9, 21]. Two additional categories that allow all scanpaths to be identified are “mixed” and “other.” The most popular strategies used by expert ATCs are circular and then linear [9, 21], and as previously explained, they are also shape dependent and are the easiest to identify. Thus, for purpose of simplification, this work divides all scanpath patterns into circular, linear, mixed, and other categories which are briefly defined below and previously illustrated in Table 1. Trajectory scans are not included because they are utilized by novices [9], and this research studies expert ATC behavior:

- *Circular*: circular scanpaths rotate in a clockwise or counterclockwise motion and include spirals and rectangles. They move along adjacent edges of the screen and tend to end adjacent from where they began.
- *Linear*: linear scanpaths are directional from one side to its opposite and move in zigzags perpendicular to their horizontal, vertical, or diagonal direction. They tend to end opposite from where they began.
- *Mixed*: mixed scanpaths occur when both circular and linear scanpaths occur and can include overlap.
- *Other*: other scans lack confident identification and therefore include patterns that are unknown or categories too difficult to confidently identify including regional, augmented, density-based, and proximity-based scanpaths.

### 2.2. Characterization Procedure

**Figure 5:**
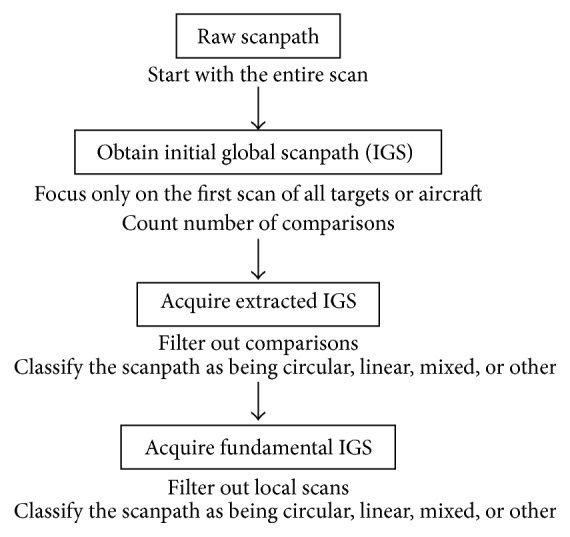
Flow chart for IGS analysis procedure.

Based on the definitions in Section 2.1, procedures were developed for identifying the number of comparisons in an IGS and for pattern classification of extracted and fundamental IGSs. The analysis process included counting the number of comparisons in the IGS, then simplifying the IGS to extracted and fundamental forms, and classifying those simplified scanpaths as circular, linear, mixed, or other as indicated in Figure 5.

Procedures were followed to determine the number of comparisons and the type of scanpath. The procedures are meant to be used on IGSs; Procedure 1 analyzes the IGS, and Procedures 2–4 analyze the fundamental IGS. Procedures 2–4 only serve as guidelines for extracted IGSs due to the many fluctuations caused by considering local scans; interrater agreement is still the dominant classification method for extracted IGSs. For fundamental IGSs, the procedures are followed for initial classification of scanpaths, and then interrater agreement is utilized to reassign pattern classification to exceptional cases that are judged incorrectly classified by the procedures.

Procedure 1 was used to count the number of comparisons made during the IGS. When aircraft were viewed for the second time, it was not counted as a comparison because it was possible that the ATC forgot a piece of information or needed to make a confirmation. When the aircraft were immediately observed for a third time, it was assumed that the ATC was making a comparison. In reference to Figure 2, following Procedure 1 leads to no comparisons in (a) and the beginning of the first comparison in (b) although aircraft *Y* was scanned 3 times in both examples.

An example is provided that applies Procedure 1 in order to increase clarity. Consider a 20-step sequence between 6 aircraft: A, B, C, D, E, and F. The sequence is as follows:

 E→A→B→C→E→F→B→E→B→D→B→E→A→B→A→B→E→A→E→C.

Table 2 shows how each aircraft appropriately falls into the states introduced in Procedure 1. In the first step, aircraft (E, A, B, C) are viewed for the first time and placed into* Scanned*; then an aircraft (E) is seen again and copied into* Potential Comparison*. In the second step, a new aircraft is viewed (F) and placed into* Scanned*, so the states* Potential Comparison* and* Comparison* clear. In the third step, aircraft from* Scanned* are viewed again (B, E) and copied into* Potential Comparison*. When an aircraft in* Potential Comparison* is additionally viewed (B), it is copied into* Comparison* where aircraft are allowed to repeat, and *N*_Comp_ is incremented to 1 (0 + 1 = 1). In the fourth step, a new aircraft is viewed (D) and placed into* Scanned*, and* Potential Comparison* and* Comparison* are cleared. In the fifth step, previous aircraft are viewed (B, E, A) and copied into* Potential Comparison* until they are repeated and therefore copied into* Comparison* (B, A, B, E, A, E) where the comparison continues between those aircraft that are also in* Potential Comparison*, and *N*_Comp_ is incremented to 2 (1 + 1 = 2). In the sixth step, an aircraft that was not in* Potential Comparison* is viewed, so* Potential Comparison* and* Comparison* are cleared. In the seventh step, since the next aircraft (C) was already in* Scanned*, it is placed in* Potential Comparison*. The number of comparisons incremented until *N*_Comp_ = 2 for this sequence.

After Procedure 1 is completed for total number of comparisons during the IGS, the extracted and fundamental IGSs are used for Procedures 2–4 for pattern classification. The procedures are general guidelines for classifying extracted IGSs but are closely followed for fundamental IGSs; they are applied by raters; then patterns are determined by interrater agreement. Scans are labeled as being circular or linear when at least 50% of the *N* total aircraft sequentially follow that pattern, because if a pattern is used on at least half of the aircraft (in the studied scenarios which range from 12 to 20 aircraft on the display), it is most likely not coincidental. Note that the percentage threshold can be adjusted and there is some allowance for points to deviate from the procedures without interrupting the sequential count of aircraft following a pattern; the aircraft count can be paused for 1 or 2 deviation points and then continued for the next aircraft if they continue the pattern. Circular and linear scans depend on the shape made by the scanpath and are independent of ATC intention. If the radar screen is represented by a grid divided into sections, the shape-dependent patterns can be conceptually identified based on the order the aircraft were observed near the outside border of the grid. Hence the center of the grid should be considered a region of error.

Circular patterns are basically achieved when an imaginary bar originating from the center of the display stretching out to the border rotates at least 180°. As the bar rotates, the scanpath must hit the aircraft it touches; circular patterns are made when the scanpath moves to adjacent points along the border, resulting in clockwise or counterclockwise movements as defined in Procedure 2. They usually result in rotating back to the starting point although that is not always the case.

Similarly, linear patterns can be conceptualized by imagining a bar that stretches across the display either vertically, horizontally, or diagonally. As the bar moves from one side to its opposite, the scanpath must hit the aircraft in contact with the bar which results in zigzag motions perpendicular to the direction the bar is moving in. Procedure 3 demonstrates this idea; the scan moves from one side or corner of the grid to the opposite, similar to wave propagation. The scanpath travels perpendicular to the direction of movement in zigzagging motions which creates switching of opposite positions along the border.

Procedures 2–4 provide conceptual frameworks to classify the patterns with chosen thresholds. The classification process is not complete until Procedures 2–4 are applied to each scanpath sequentially. Certain assumptions are made before utilizing Procedures 2 and 3: there is a uniform distribution of aircraft across the display, tolerance is applied by raters to allow some deviation from the ideal patterns, and the procedures are based on assuming that the spatial layout of the multiple targets (or aircraft) are distributed in a uniform manner with random spacing, meaning that they are not equally aligned following a uniform distribution.

**Figure 6:**
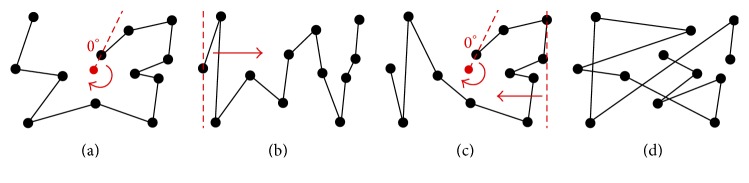
Fundamental scanpath examples that are identified using Procedures 2–4. (a) Circular scanpath identified with Procedures 2 and 4 (Step 1). (b) Linear scanpath identified with Procedures 3 and 4 (Step 2). (c) Mixed scanpath identified with Procedure 4 (Step 3). (d) Another scanpath identified with Procedure 4 (Step 4).

The rationale for Procedure 2 is as follows. The angle of the first eye fixated target is always set at *θ*_1_ = 0°. Step 1 assigns *θ* values to each subsequent eye fixated target in reference to *θ*_1_ (e.g., in Figure 6(a), *θ*_2_ is 15° clockwise from *θ*_1_). Steps 2 and 3 investigate whether there is a consistent increase or decrease of *θ* values. If increasing, then the visual scan is in a clockwise motion, and if decreasing, then the visual scan is in a counterclockwise motion. Note that for a counterclockwise movement, *θ*_1_ (where *i* = 1) is followed by *θ*_*k*_ (where *i* = *k*), and then the *i* values decrease from *k*. In Step 4, if the identified number of eye fixated targets (*n*) is over half the total eye fixated targets (*N*), then we classify the scanpath as circular. Note that the threshold used in Step 6 can be adjusted. A tolerance in sequential increase or decrease must be allowed (e.g., a few *θ*_*i*_ values can increase among an overall decrease of *θ*_*i*_ values) since eye movements are not mechanical and often deviate from ideal mathematical patterns or verbal explanations of individual's search patterns. Examples of circular scanpaths classified by Procedure 2 are shown in Figures 6(a) and 6(c). Each black point represents a target; the first target being fixated on is 0° (*θ*_1_). In Figure 6(a), the remaining points have sequentially increasing *θ* values which creates a clockwise rotation. In Figure 6(c), the first 10 points apply to a circular pattern, which are over half of the aircraft; therefore it also satisfies Procedure 2.

The rationale for Procedure 3 is as follows. Step 1 defines the starting point of the aircraft fixated on an extreme location on the *x*- or *y*-axis of the display. The starting point does not need to be the first eye fixation point on the target since the pattern may not start until a few eye fixations occurred on other targets. Also note that *X* and *Y* extrema can include any points relatively near the outside border of the screen to allow some tolerance; they do not have to be the absolute extrema, but they need to be close. Steps 2–5 define the general trend of linear movements of either vertical or horizontal movements. In detail, the movements can be in the overall vertical direction with horizontal zigzags or in the overall horizontal direction with vertical zigzags. The reason that there are 4 differentiated steps is that each step indicates whether the overall direction is increasing from left to right (Step 2), right to left (Step 3), bottom to top (Step 4), or top to bottom (Step 5) and insures they end near the opposite side of the display. In Step 6, if the identified number of eye fixated targets (*n*) is over half the total eye fixated targets (*N*), then we classify the scanpath as being linear. Note that the threshold used in Step 6 can be adjusted. Again, a tolerance in the overall direction must be allowed (e.g., a few *X*_*i*_ (or *Y*_*i*_) values can increase among overall decrease of *X*_*i*_ (or *Y*_*i*_) values). Examples of linear scanpaths classified by Procedure 3 are shown in Figures 6(b) and 6(c). The scanpath in Figure 6(b) starts at *X*_min_, then the *X* values consistently increase as the *Y* values switch directions (between increasing and decreasing) 6 times, and finally it ends at *X*_max_; the linear direction is horizontal from left to right. In Figure 6(c), the last 10 points apply towards a horizontal linear pattern from right to left (the third point is excluded, so 9 total points count towards the linear pattern), which include over half of the aircraft; therefore it also satisfies Procedure 3.

The rationale for Procedure 4 is as follows. Steps 1 and 2 cause the scanpath to be analyzed for circular or linear patterns. If the scanpath is exclusively circular or linear, it is classified as such. In Step 3, if the scanpath can satisfy both requirements for circular and linear patterns, it is labeled mixed. Step 4 is provided to assign “other” classification for scanpaths that do not utilize circular or linear patterns at all. After the procedures are followed, interrater agreement is used in order to account for exceptional cases. Utilizing interrater agreement is a necessity due to the fact that realistic scanpaths do not follow ideal mathematical patterns. All of the figures provided in Figure 6 are classified using Procedure 4. Part (a) is classified by Step 1, part (b) is classified by Step 2, part (c) is classified by Step 3, and part (d) is classified by Step 4. The example in (c) is mixed because the first 10 fixations qualify as being circular since the *θ* values are increasing, and the last 10 fixations (excluding the third point) qualify as being linear since the *X* values are consistently decreasing as the *Y* values switch direction twice. The example in (d) is “other” because neither circular nor linear patterns occur consecutively for at least half of the aircraft.

## 3. Experiment

### 3.1. Participants

At Indianapolis ARTCC, 25 expert ATCs with FAA certification provided the scanpaths. Their experience ranged from 3 to 30 years with an average of 20.7 and standard deviation of 7.1. Due to too much loss of data to draw confident conclusions, 1 participant was excluded from each scenario resulting in the analysis of 24 participants across 3 scenarios for a total of 72 recordings.

### 3.2. Apparatus

A Tobii X60 eye tracker was used to collect the eye tracking data of the participants at a collection rate of 60 Hz. Simscope/Simtarget software was used to simulate a radar display of air traffic on a 48.26 cm LCD monitor. The eye tracker had an accuracy of 0.5° with each degree corresponding to approximately 1.2 cm when eyes were 68.6 cm from the monitor. Participants' eyes were about 100 cm from the monitor which resulted in a maximum fixation error of 1 cm. Data blocks were 1.5 cm by 1.1 cm; therefore the data block visual angle was about 0.6° due to the height. Digital surveillance radar (DSR) mode was used on Simscope/Simtarget with a refresh rate of 5 s, which simulates realistic radar displays with considerable accuracy.

### 3.3. Task

ATCs had to identify and solve conflicts while their eye tracking data was collected. Before the tasks began, 2 practice scenarios were performed in order to familiarize the ATCs with the simulation. During the following tasks that were recorded for data analysis, they were required to announce aircraft call signs of LOS pairs until no more remained during 3 unique scenarios.

### 3.4. Scenarios

**Figure 7:**
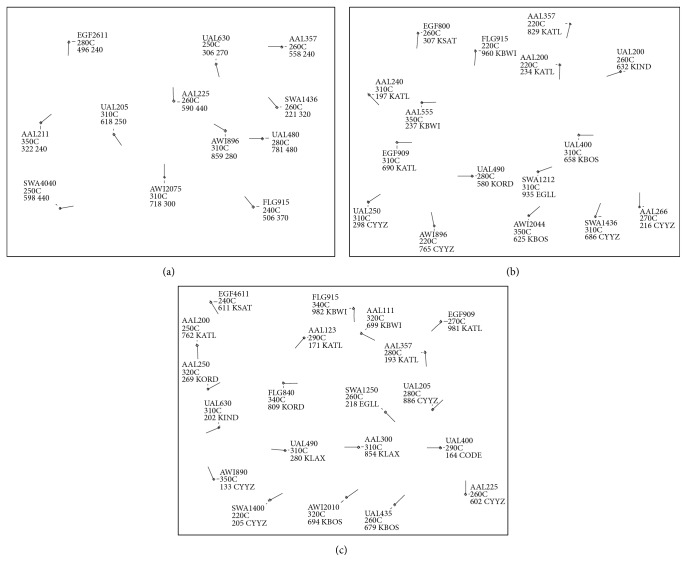
Scenarios performed by each participant. (a) Low congestion scenario. (b) Moderate congestion scenario. (c) High congestion scenario.

There were 3 en route scenarios presented to each participant that are displayed in Figure 7: (1) low congestion scenario with 12 aircraft shown in (a), (2) moderate congestion scenario with 16 aircraft shown in (b), and (3) high congestion scenario with 20 aircraft shown in (c). The simulations have black backgrounds with bright green objects and text, but to enhance the images, the color was inverted and converted to black and white, and the text size was increased by 200%. Each small diamond shape symbolizes an aircraft and the line projecting out indicates the direction of travel. The display provides a top-down view of the aircraft as if they are being observed from above, looking down towards the Earth's surface. The three lines of text by each aircraft is the data tag which lists the flight number, altitude, and speed, respectively. The aircraft in these scenarios are en route; hence each has constant altitudes indicated by the “C” following the altitude.

### 3.5. Data Analysis

The independent variable was the aircraft congestion of low, moderate, and high. The dependent variable was each resulting scanpath. From each raw scanpath, the IGS was obtained and then extracted and fundamental IGSs were obtained. The raw scanpath was measured for total time, the IGS was analyzed for scan time and number of comparisons by applying Procedure 1, and the extracted and fundamental IGSs were analyzed for pattern classification of circular, linear, mixed, or other using Procedures 2–4 with interrater agreement.

Tobii Studio software was used to collect and analyze the eye tracking data. The velocity threshold identification (I-VT) algorithm [34, 35] was used with the defaulted threshold of 0.42 pixels/ms to define a spatial fixation.

Analysis of oculomotor trends included average raw scanpath time versus aircraft congestion, average IGS time versus aircraft congestion, average number of IGS comparisons versus aircraft congestion, and average IGS time versus average number of IGS comparisons. The results were plotted and an ANOVA test was applied with pairwise comparisons between each relationship.

For visual scan pattern classification, two raters utilized Procedures 2–4 to reach an interrater agreement. Extracted scanpath classification is similar to the previous method used in [9] because interrater agreement was dominant and the procedures were only used as guidelines. Fundamental scanpaths were classified using a more elaborate procedure than what was previously used in [9]. Classification depended on the procedures; then interrater agreement was used to confirm accurate use of the procedures and reassign classification to exceptional scanpaths that were judged incorrectly classified by the procedures. Each scan was reviewed at least 3 times by the raters to minimize judgement errors. The results of extracted and fundamental scanpath patterns were compared against each other to see if they differed and with ATCs' verbal inputs from [21] to check consistency.

## 4. Results

From all 75 recordings, 3 were excluded due to missing periods of eye tracking data: participant 5 in low and moderate congestion scenarios and participant 22 in high congestion scenario. Therefore 72 recordings were used, 24 recordings from each scenario.

### 4.1. Oculomotor Trends

**Figure 8:**
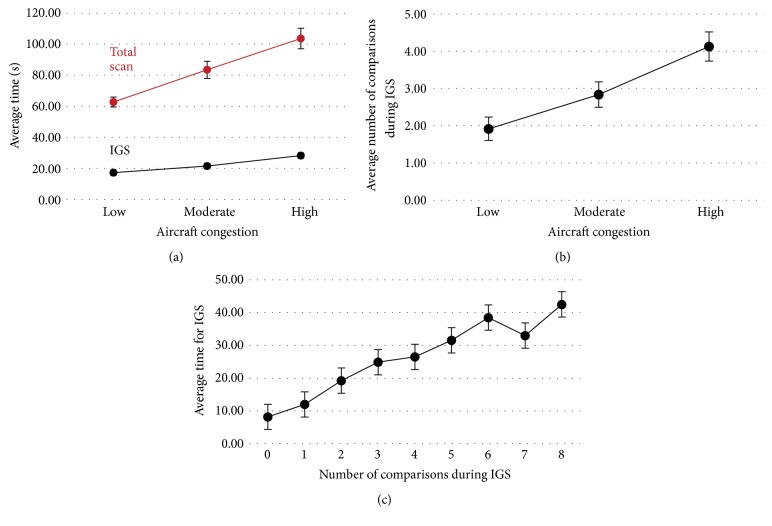
Oculomotor trends. (a) Average time to complete the raw scanpath (red) and average time to perform the IGS (black). (b) Number of comparisons during the IGS. (c) Average time to complete the IGS with respect to the identified number of comparisons during the IGS.

The oculomotor trends include scan time and number of comparisons as aircraft congestion increases. Figures 8(a) and 8(b) illustrate how scan time and number of comparisons both increased with more aircraft. As ATCs conducted more comparisons, they took longer to complete the IGS as shown in Figure 8(c), which implies that increasing the amount of aircraft increases the number of comparisons which increases the required scan time.

For the following data analysis on oculomotor trends, *α* = 0.05 was used to determine significance of results. The different congestion levels (low, moderate, and high) had significant effect on the total scan time (*F* = 5.47, *p* < 0.001) illustrated in Figure 8(a). Post hoc analysis (Tukey test) showed that there were significant differences among all congestion levels (*p* < 0.001 for all pairwise comparisons). Similarly, the different congestion levels had significant effect on the IGS time (*F* = 4.14, *p* < 0.001). Post hoc analysis (Tukey test) showed significant differences for low versus high (*p* < 0.001) and moderate versus high (*p* < 0.001), and marginal differences for low versus moderate (*p* = 0.08). The different congestion levels had significant effect on the mean number of comparisons (*F* = 2.53, *p* < 0.001) illustrated in Figure 8(b). Post hoc analysis (Tukey test) showed significant differences for low versus high (*p* < 0.001) and moderate versus high (*p* = 0.010), and insignificant differences were found for low versus moderate (*p* = 0.108). The number of comparisons had significant effect on the IGS time (*F* = 11.80, *p* < 0.001) illustrated in Figure 8(c). Post hoc analysis (Tukey test) showed significant differences for most pairwise comparisons, as depicted in Table 3.

### 4.2. Scanpath Patterns

**Figure 9:**
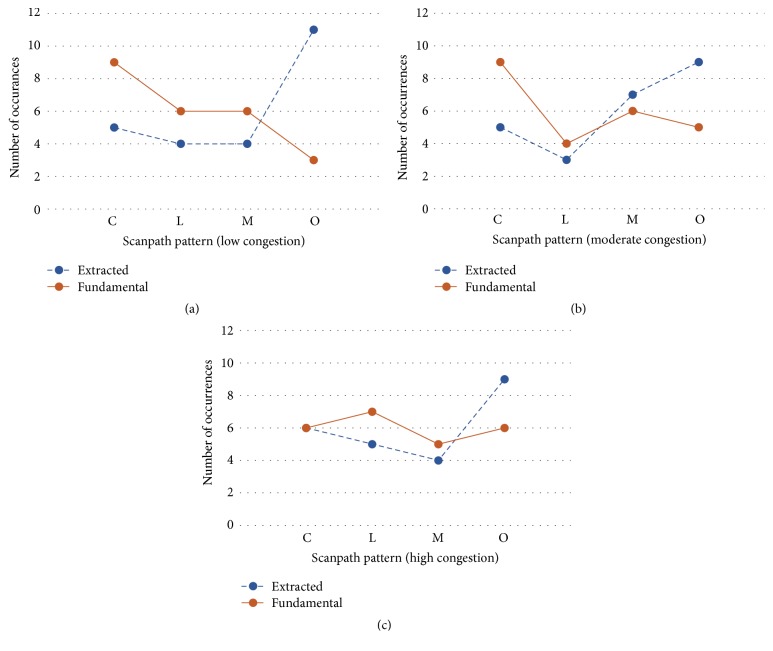
Number of participants classified with given scanpath patterns for extracted IGS (blue dashed line) and fundamental IGS (orange solid line). (a) Low congestion scenario. (b) Moderate congestion scenario. (c) High congestion scenario.

The results of the extracted and fundamental scanpath classifications for different levels of congestion are provided in Figure 9. The number of participants to use the given patterns is shown for circular (C), linear (L), mixed (M), and other (O). Several trends can be witnessed from the data. The fundamental scanpaths show a similar trend for low and moderate congestion if mixed patterns are not considered (since it is unknown if they should be counted as circular, linear, or other): circular scanpaths are most common, followed by linear, and other scanpaths are least common. For high congestion, the pattern occurrences are fairly consistent. However, for the extracted scanpaths, the trends were quite different. Other scanpaths were most common due to the influence of local scans, and they in fact consist of almost half of the identified patterns. Circular patterns are slightly more occurrent than linear, but neither is frequent, and mixed patterns are least popular except in the moderate congestion scenario where they suddenly rise.

The detailed scanpath patterns identified from the extracted and the fundamental scanpaths of all participants during the IGS are shown in Table 4 for each scenario. As previously explained, the “extracted scanpath pattern” was determined by observing the IGS while excluding comparisons. The “fundamental scanpath pattern” was derived from the shape created by the order the aircraft were viewed, which excluded any repeated fixations. The fundamental scanpath is the IGS without local scans (which also excludes comparisons); consequently the scanpath does not return to aircraft already scanned. Usually the patterns observed in the fundamental scanpaths were the same or simpler than the extracted patterns, although that was not always the case; there were 3 exceptions (low scenario participant 20, moderate scenario participant 18, and high scenario participant 23). N/A indicates substantial loss of eye tracking data not included in the analysis.

**Figure 10:**
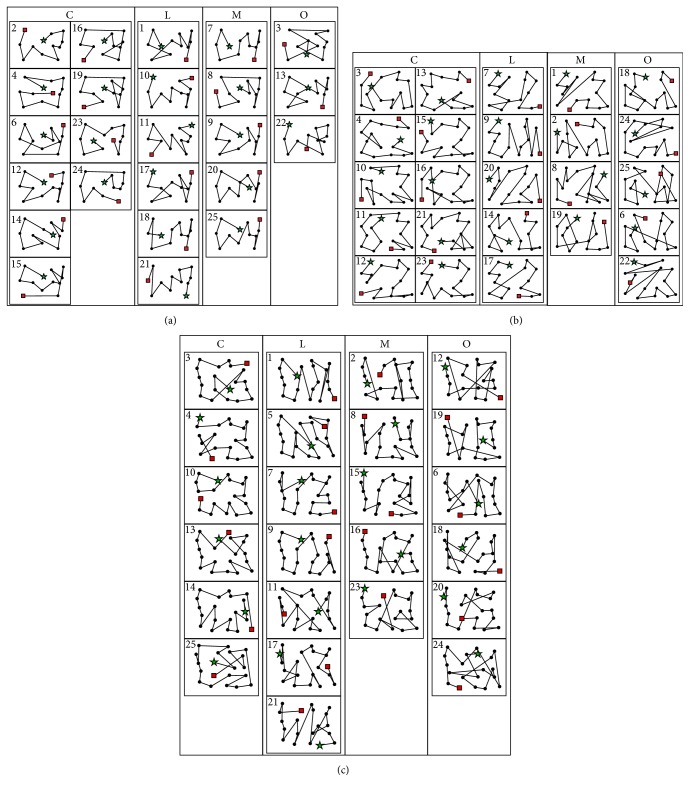
Illustrated fundamental IGSs grouped in their pattern classifications. (a) Low congestion scenario. (b) Moderate congestion scenario. (c) High congestion scenario.

Figure 10 illustrates the fundamental scanpaths. Each black dot represents an aircraft and the starting and ending aircraft are marked with a green star and red square, respectively. They are grouped into the categories exactly as indicated from Table 4 (C, L, M, or O) and the participant number is on the top left of each scanpath. The classification was chosen based on Procedures 2–4 provided above and then interrater agreement to reassign any exceptions. Note that the fundamental scanpaths are a still image drawn based on the initial location of each aircraft to provide better insights to the visual patterns. In reality, each aircraft is in movement during the IGS of ATCs. However, the movement of each aircraft was substantially small during the IGS (e.g., approximately 95 pixels or 0.25 cm of movement on the display every 5 seconds when flying at 300 knots). Therefore, it was determined that it was sufficient to use a single figure with initial aircraft location overlaid with the scanpath, rather than having to show multiple screenshots of different aircraft locations during the IGS.

**Figure 11:**
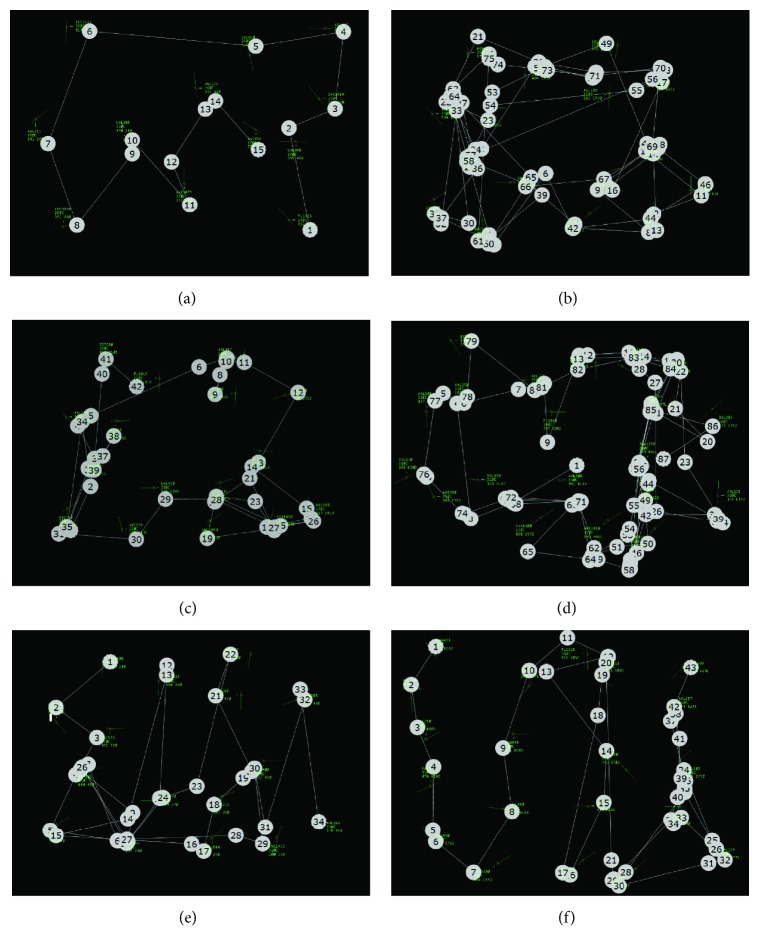
Examples of global scanpaths overlaid on the radar display. (a) Circular scanpath in low congestion scenario. (b) Circular scanpath in moderate congestion scenario with three complete revolutions. (c) Circular scanpath in moderate congestion scenario. (d) Circular scanpath in high congestion scenario. (e) Linear scanpath in moderate congestion scenario. (f) Linear scanpath in high congestion scenario.

Extracted scanpaths are not graphically displayed because they depended on the raters' cognitive ability of watching the IGS and disregarding any comparisons, similar to the previous method used for identifying scanpath patterns. Nonetheless, examples of identified global scanpaths from collected data are shown in Figure 11 when definite circular or linear patterns occurred. The aircraft and data tags are in green, the white numbered circles show the sequential eye fixations, and each line represents a saccade between fixations. Rater judgement was made on extracted scanpaths by observing the IGSs similar to global scanpaths displayed in Figure 11, cognitively neglecting comparisons, and then determining the classification based on the scanpath pattern.

The obtained results were compared to the ATCs' linguistic inputs from [21] in Table 5. The verbal inputs were applied to 26 low congestion scenario cases and only consisted of circular (C), linear (L), or other (O) patterns. The fundamental and extracted pattern results were applied to all scenarios and consisted of 24 cases each, with an additional mixed (M) category. As the table indicates, the low congestion fundamental patterns are most consistent with the ATCs' inputs. The occurrences from the results were expected to differ because of the added presence of a mixed category, but most of the trends are similar. If the mixed category was removed causing mixed scanpaths in the low scenario of fundamental patterns to instead be classified as 5 other patterns and 1 circular pattern, those findings would match the verbal inputs as much as possible given the unequal participant numbers.

## 5. Discussion

The scanpaths were characterized based on (1) oculomotor trends including raw scanpath completion time, IGS time, and number of comparisons for differing aircraft congestion scenarios and (2) extracted and fundamental IGS patterns. Not all of the patterns that ATCs self-reported in [21] were identified, although similar patterns were found from observing the scans, without considering the linguistic input from the ATCs.

The oculomotor trends in Figure 8 showed that there were significant differences between congestion levels when examining total raw scanpath time or initial global scanpath (IGS) time. The scan times significantly increased as the congestion increased; however the amount of increase in the IGS time based on congestion was not proportionally linear to the amount of increase in total scan time. It appears that the ATCs took much more time to detect the aircraft conflicts as the congestion level increased but tried to complete the IGS as quickly as possible. This reasoning is supported by the average rate of increase of the total raw scanpath time being higher than the average rate of increase of the IGS time. This is most likely due to the average number of comparisons during the IGS moving from only 2 (low congestion) to 3 (moderate congestion) to 4 (high congestion) comparisons; only 1 more comparison was used each time the congestion scenario increased, but the total number of comparisons probably increased much more across the scenarios which resulted in the total scan time rate increasing much faster than the IGS rate.

The visual scanning strategies show that the most dominant patterns used by expert ATCs were circular method followed by linear method, followed by other methods, which accords with the ATCs' linguistic inputs that were provided in [21]. An important finding was that the fundamental scanpaths showed more consistent matching with the ATCs' linguistic inputs compared to those identified from the extracted scanpaths. In Figure 9, similar trends can be viewed between circular, linear, and mixed patterns, but extracted scanpaths have much more “other” patterns than fundamental scanpaths. This indicates that the “other” patterns in extracted scanpaths were classified as being either circular, linear, or mixed in the fundamental scanpaths. When observing the individual trends in Table 4, approximately 68% of the classifications were identically matched between those scanpaths, and the remaining 32% of pattern classifications consisted of the mentioned differences between the scanpaths.

Interestingly, there was a slight increase in the frequency of the linear search patterns when the congestion level was high, and possible reason for that could be due to the initial spatial layout of the multiple aircraft in that scenario which seemed to be dominantly linear. Based on the multiple observations of the ATCs' visual scanning patterns, the ATCs seem to apply an overall scan pattern (such as circular or linear), but it also seems that they move from one aircraft to another in close (or closest) proximity. If the spatial layout is somewhat linear, then even if an ATC has a circular search strategy in mind, the visual scanpath may result in a linear pattern and that could explain the higher number of linear patterns used in the high congestion scenario that can be seen Figure 9(c).

Another explanation could be that the ATCs may have changed their strategies from circular to linear as the congestion level increased. It may have been easier for the ATCs to use a linear scanning strategy to keep track of the observed aircraft as the scenarios became more complex. Individual scanpath pattern comparisons in Table 4 show that many ATCs were consistent with their visual scanning strategies across the scenarios (e.g., participants 4, 23, and 17); however, some ATCs showed different patterns among different congestion levels (e.g., participants 10, 11, and 25). However, the amount of change from circular to linear was not drastic, and it is challenging to identify the possible reasons of the individual inconsistencies by only examining the scanning patterns.

Figure 10 illustrates the categorized fundamental IGSs that were judged and agreed upon the interraters based on the developed processes. The result shows that scans can be classified by simply observing their filtered representations and applying definitive procedures without any follow-up validations by the ATCs. The obtained results could not be 100% mapped to the ATCs' linguistic inputs but showed high promise with similar mapping percentages, as shown in Table 5. Note that perfection was not expected since the results included an extra classification category (mixed) as opposed to those verbally expressed by ATCs, but the trends are similar in which circular patterns are more popular than linear patterns.

As previously mentioned, fundamental and extracted patterns differ mainly in the other category; other pattern is the most popular pattern for extracted scanpaths, but they are less frequent than circular patterns for fundamental scanpaths. The mapping percentages were closest to ATCs' inputs for the fundamental scanpath during the low congestion scenario. If the 6 occurrences that were judged mixed in that case were instead classified as 5 other and 1 circular, the trend would have matched the ATCs' inputs as much as possible (note that the ATCs' inputs total 26 while each fundamental and extracted scenario total 24). In fact, if all 6 of the mixed patterns were classified instead as other, the low congestion fundamental case would be consistent with the inputs provided in [21]. This consistency indicates promise in utilizing the classification method on fundamental scanpaths.

*Limitations and Future Directions*. Scanpath patterns used by ATCs can be unintended, incomplete, and limited to local scans, or overlapping with other categories which makes classification challenging. Circular and linear scans were the easiest to identify because they are independent of the above limitations mentioned; they depend on shape and follow certain procedures. However, regional, augmented, density-based, and proximity-based scans are considered difficult to identify since they do not depend on shape. At this time, they lack confident identification from the eye tracking data alone unlike circular and linear scans; therefore they hold too much uncertainty for current identification and are classified as other scans. Other and mixed scans are subjectively the most difficult to recognize, with other scans ranking as most difficult. Although the remaining patterns increase in classification difficulty, it remains useful to develop algorithms that can encapsulate all strategies mentioned by ATCs.

The effects of several variables have been studied in this work and in previous research, such as scenario congestion and difficulty. Spatial layout also needs to be investigated to determine how different layouts can influence the visual scanning patterns and analysis of them. In an extreme case, if all aircraft were aligned into a single line, the visual scanning pattern would always be linear even if the ATC attempted a circular strategy.

Perhaps the most difficult aspect of this research was in identifying the cognitive reasons to the observed visual scanning patterns under different aircraft congestion levels. The visual scanpaths provide different types of search methods but do not necessarily show the rationale underlying the search pattern. Therefore, a mixed method approach was required to validate the classified visual scanning patterns through the ATCs' follow-up confirmation on the classified patterns.

Based on this research, it seems that the ATCs' intended strategies are composed of 3 parts: (1) aircraft are searched with a pattern to complete a global scan, (2) aircraft in potential conflict are selected with local scans, and (3) comparisons are made between aircraft to solve conflicts [21]. The order in which these steps occur and whether they overlap differ with individual ATCs. For example, some ATCs completed a global scan before using local scans and then start comparisons, and others use local scans with comparisons to eventually form their global scan. If an ATC decides how to complete each step and in which order, it may be possible to define the ATC's intended strategy leading to better mapping the visual scanpaths to each ATC's intended strategy.

The goals in analyzing expert ATC scanpaths are to (1) develop high quality training programs for novices and (2) use automation to aid ATCs as their jobs grow more difficult with increased aircraft traffic. Characterizing ATCs' strategies observed during complex and critical situations can be used to better aid novice ATCs during training. Automation can be applied in many ways, such as informing ATCs when multiple aircraft were not scanned in an effective manner or when possible conflicting aircraft were not adequately identified. The succeeding step of this research is to compare the results obtained using the procedures on fundamental scanpaths with ATCs' inputs to test methodology accuracy through implementing the procedures into robust computer algorithms. Furthermore, in the long term, we should be able to support multimodal input analysis, such as corroborating EEG analysis with eye tracking analysis [36] to better support our goals.

## 6. Conclusion

Finding similar visual scanpath patterns that map with the ATCs' linguistic inputs were accomplished by selectively using the IGS, extracting the fundamental representation, and applying Procedures 2–4 for classification that allowed less reliability on rater judgement. The development and classification of the fundamental scanpaths showed promise in better mapping the visual scanning patterns to the ATC linguistic inputs; it was found that the mixed patterns should instead be most likely classified as “other” patterns. Moreover, oculomotor trends revealed the effects of different aircraft congestion; as congestion increased, scan time and number of comparisons increased as well. Scanpath patterns were also affected by increasing aircraft congestion by a higher occurrence of “other” patterns in the fundamental scanpaths, although more studies are required to determine the cause. Improving the classification procedures and developing algorithms would be highly useful for identifying scanpath patterns more accurately. Once appropriate algorithms are generated, pattern identification can be automated and utilized in further understanding of ATC cognitive processes, effective training methods, and improvements of the ATC interface.

## Figures and Tables

**Procedure 1:**
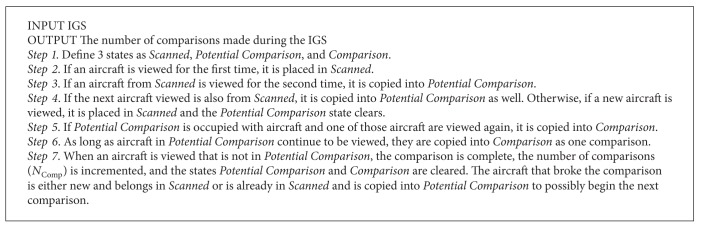
Counting the number of comparisons.

**Procedure 2:**
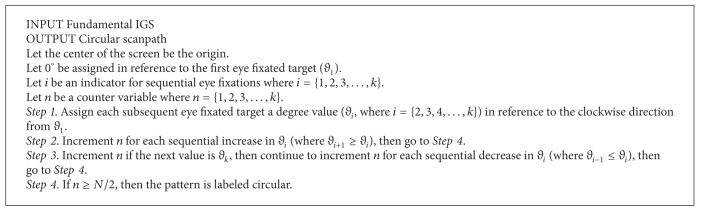
Identifying circular scanpaths.

**Procedure 3:**
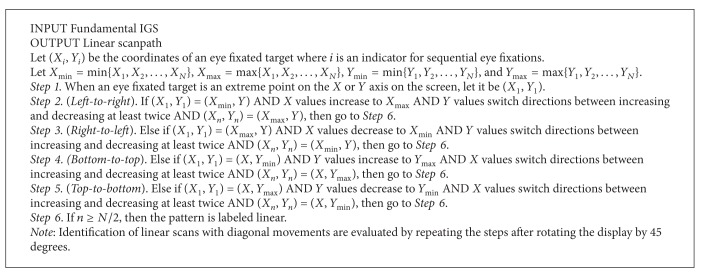
Identifying linear scanpaths.

**Procedure 4:**
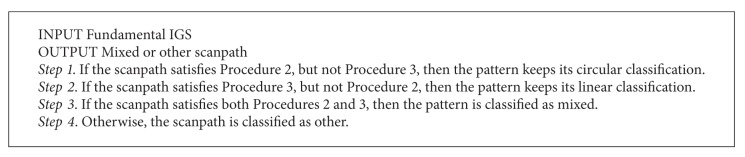
Identifying mixed and other scanpaths.

**Table 1:** Ideal and realistic examples of relevant scanpath patterns.

	Circular	Linear	Mixed
Ideal examples	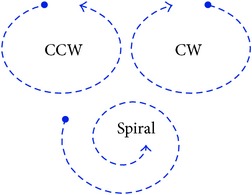	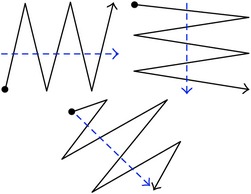	N/A

Realistic examples	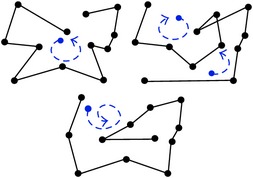	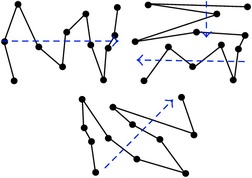	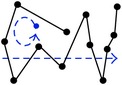

**Table 2:** Application example of Procedure 1.

Step	Scanned	Potential comparison	Comparison	*N*_Comp_
1	E, A, B, C	E	—	0
2	E, A, B, C, F	—	—	0
3	E, A, B, C, F	B, E	B	1
4	E, A, B, C, F, D	—	—	1
5	E, A, B, C, F, D	B, E, A	B, A, B, E, A, E	2
6	E, A, B, C, F, D	—	—	2
7	E, A, B, C, F, D	C	—	2

**Table 3:** Least squares means for effect number of comparisons. Pr > |*t*| for *H*0: LSMean(*i*) = LSMean(*j*). Dependent variable: IGS time.

*i*/*j*	1	2	3	4	5	6	7	8	9
1		0.234	0.010	<0.001	<0.001	<0.001	<0.001	<0.001	<0.001
2	0.234		0.966	0.043	0.003	<0.001	<0.001	<0.001	<0.001
3	0.010	0.966		0.171	0.016	<0.001	<0.001	0.001	<0.001
4	<0.001	0.043	0.171		0.973	0.038	0.006	0.041	<0.001
5	<0.001	0.003	0.016	0.973		0.400	0.030	0.094	0.001
6	<0.001	<0.001	<0.001	0.038	0.400		0.360	0.906	0.012
7	<0.001	<0.001	<0.001	0.006	0.030	0.360		1.000	0.943
8	<0.001	<0.001	0.001	0.041	0.094	0.906	1.000		0.695
9	<0.001	<0.001	<0.001	<0.001	0.001	0.012	0.943	0.695	

**Table 4:** Scanpath patterns identified during IGS.

Participant	Extracted scanpath pattern			Fundamental scanpath pattern		
	Low	Moderate	High	Low	Moderate	High
1	Other	Mixed	Linear	Linear	Mixed	Linear
2	Other	Mixed	Mixed	Circular	Mixed	Mixed
3	Other	Circular	Mixed	Other	Circular	Circular
4	Circular	Circular	Circular	Circular	Circular	Circular
5	N/A	N/A	Linear	N/A	N/A	Linear
6	Other	Other	Other	Circular	Other	Other
7	Mixed	Linear	Linear	Mixed	Linear	Linear
8	Other	Mixed	Other	Mixed	Mixed	Mixed
9	Mixed	Linear	Linear	Mixed	Linear	Linear
10	Mixed	Other	Circular	Linear	Mixed	Circular
11	Other	Circular	Other	Linear	Circular	Linear
12	Circular	Other	Other	Circular	Circular	Other
13	Other	Other	Circular	Other	Circular	Circular
14	Circular	Other	Circular	Circular	Mixed	Circular
15	Other	Circular	Mixed	Mixed	Circular	Mixed
16	Other	Mixed	Mixed	Circular	Circular	Mixed
17	Linear	Mixed	Linear	Linear	Linear	Linear
18	Linear	Mixed	Other	Linear	Other	Other
19	Other	Mixed	Other	Circular	Mixed	Other
20	Linear	Linear	Other	Mixed	Linear	Other
21	Linear	Other	Other	Linear	Circular	Linear
22	Other	Other	N/A	Other	Other	N/A
23	Circular	Circular	Circular	Circular	Circular	Mixed
24	Circular	Other	Other	Circular	Other	Other
25	Mixed	Other	Circular	Mixed	Other	Circular

**Table 5:** Pattern occurrence from ATCs' linguistic inputs compared to fundamental and extracted scanpaths.

	ATCs' inputs		Fundamental patterns						Extracted patterns					
	Low		Low		Moderate		High		Low		Moderate		High	
C	11	42%	9	38%	9	38%	6	25%	5	21%	5	21%	6	25%
L	6	23%	6	25%	4	17%	7	29%	4	17%	3	13%	5	21%
M	N/A	N/A	6	25%	6	25%	5	21%	4	17%	7	29%	4	17%
O	9	35%	3	13%	5	21%	6	25%	11	46%	9	38%	9	38%
